# Oral health‐related habits and behaviors and risk for cognitive decline and incident dementia

**DOI:** 10.1002/alz70860_097662

**Published:** 2025-12-23

**Authors:** Sam Asher, Alina Solomon, Ruth Stephen, Tiia Ngandu, Seppo Koskinen, Anna Liisa Suominen

**Affiliations:** ^1^ Institute of Dentistry, University of Eastern Finland, Kuopio, Finland, Kuopio, Finland; ^2^ Imperial College London, London, United Kingdom; ^3^ Karolinska Institutet, Stockholm, Sweden; ^4^ Institute of Clinical Medicine, University of Eastern Finland, Kuopio, Finland; ^5^ Finnish Institute for Health and Welfare, Helsinki, Finland; ^6^ Department of Clinical Medicine/Neurology, University of Eastern Finland, Kuopio, Finland, Kuopio, Finland; ^7^ Kuopio University Hospital, Kuopio, Finland

## Abstract

**Background:**

Literature shows a higher dementia risk for individuals with oral diseases. However, subjective aspects of oral health, habits, and behaviors, are yet to be considered in relation to long‐term impact on cognition.

**Method:**

Data from Finnish Health 2000 and its follow‐up Health 2011 surveys was used. Oral health variables included daily brushing frequency (≥2, once, <1), frequency of dental visits in past 12 months (0, 1‐2, ≥3), reason for availing dental healthcare (periodically/on experiencing symptoms), experiencing tooth/denture related problem in past 12 months (no/yes), self‐reported oral health (good/not‐good), and xerostomia (yes/no). Across different oral health variables sample size varied: 11‐year cognitive decline *N* = 2962‐3276; 15‐year incident dementia, *N* = 4409‐5130.

Cognitive assessment at baseline and follow‐up included verbal fluency, total immediate and delayed recall. To assess overall cognition, we calculated composite cognitive score‐CCS as sum of individual tests. For each test including CCS, cognitive decline over 11‐years was calculated as difference in H2011 and H2000 scores (decline/no decline). ICD‐codes for dementia diagnosis until 2015 was retrieved from national health registers. Analysis included binary logistic and Cox proportional regression and adjusted for relevant factors.

**Result:**

After full adjustments, brushing <1 times and ≥3 dental visits in past 12 months was associated with total immediate recall decline, availing dental healthcare only on experiencing symptoms with verbal fluency decline and having xerostomia with delayed recall decline. For 15‐year incident dementia, higher risk was observed for individuals having ≥3 dental visits in past 12 months, experiencing tooth/denture related problem in past 12 months, and having not‐good self‐reported oral health.

**Conclusion:**

Poor oral health habits, recent history of oral health problems, and extensive use of dental healthcare have long‐term adverse impact on cognition. Studies into underlying mechanisms and impact of oral health interventions are warranted.

